# Laboratory Testing Methods for Novel Severe Acute Respiratory Syndrome-Coronavirus-2 (SARS-CoV-2)

**DOI:** 10.3389/fcell.2020.00468

**Published:** 2020-06-04

**Authors:** Roshan J. D'Cruz, Arthur W. Currier, Valerie B. Sampson

**Affiliations:** Nemours/Alfred I. duPont Hospital for Children, Wilmington, DE, United States

**Keywords:** coronavirus, RT-PCR, EIA, lateral flow diagnostics, convalescent plasma

## Abstract

Following the first reports of coronavirus disease-19 (COVID-19) by China to the World Health Organization (WHO) on 31st December 2019, more than 4,302,774 novel severe acute respiratory syndrome coronavirus-2 (SARS-CoV-2) cases have been reported by authorities in 212 countries and territories by 12th May 2020. The outbreak and spread of COVID-19 worldwide, highlights the critical need for developing rapid and accurate diagnostic testing methods for emerging human coronavirus (CoV) infections. Testing is crucial to track the spread of disease during a pandemic, and to swiftly permit public health interventions including isolation, quarantine, and appropriate clinical management of afflicted individuals. The key components of viral diagnostic tests are (1) collection of the appropriate sample (blood, nasal swab, and throat swab), (2) availability of the genetic and proteomic sequences of the novel virus for analysis, and (3) rapid and accurate laboratory testing methods. The current gold standard for the molecular diagnosis of SARS-CoV-2 infection is the real-time reverse transcriptase-polymerase chain reaction (RT-PCR) for the qualitative and quantitative detection of viral nucleic acids. Other relevant laboratory methods include enzyme-linked immunoassays (EIA) for viral antibody and antigen detection, and serum viral neutralization (SVN) assays for antibody neutralization determination. The challenges faced in developing a diagnostic test for a novel pathogen are the ability to measure low viral loads for early detection, to provide low or no cross-reactivity with other viral strains and to deliver results rapidly. Several point-of-care molecular devices are currently being integrated for fast and accurate diagnosis of SARS-CoV-2 infections. This review discusses the current laboratory methods available to test for coronaviruses by focusing on the present COVID-19 outbreak.

## Introduction

Coronavirus disease-19 (COVID-19) is caused by a novel coronavirus (CoV) that was originally reported in Wuhan, Hubei province, China in December 2019 (World Health Organization, 2020a). The International Committee on Taxonomy of Viruses named the virus severe acute respiratory syndrome coronavirus 2 (SARS-CoV-2). Infection by SARS-CoV-2 causes a respiratory illness that varies in severity from mild upper respiratory symptoms akin to the seasonal flu, to severe progressive respiratory failure that requires intensive care and can lead to death. Asymptomatic carriers of the virus have also been reported and pose a significant public health threat due to their ability to unknowingly spread the virus (Chan et al., 2020a). SARS-CoV-2 represents the third CoV in this millennium to cross species from animals to humans and cause a severe respiratory disease after Middle-East respiratory syndrome coronavirus (MERS-CoV) in 2012 (Zaki et al., 2012), and SARS-CoV in 2003 (Drosten et al., 2003; Ksiazek et al., 2003). This novel CoV has now been identified as the seventh CoV that is transmissible between humans (including HCoV-229E, HCoV-OC43, HCoV-NL63, and HCoV-HKU1) (Salata et al., 2019). On 30th January 2020, the World Health Organization (WHO) declared the SARS-CoV-2 epidemic a public health emergency of international concern and was upgraded to a pandemic on 11th March 2020. At least 4,302,774 confirmed cases and 289,561 deaths worldwide were reported as of 12th May 2020 (worldometers.info/coronavirus/). Diagnostic testing is critical during a pandemic as the ability to track the spread of SARS-CoV-2 is essential for effective disease management and control.

**Figure 1:**
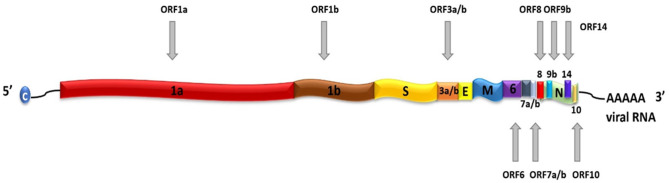
Schematic representation of SARS-CoV-2 genome. SARS-CoV-2 contains a positive-sense, positive-stranded mRNA genome with a 5′ capped mRNA sequence (C) and a 3′ poly-A tail. The coding genes are: ORF1a, ORF1b, Spike (S), ORF3a, ORF3b, Envelope (E), Membrane (M), ORF6, ORF7a, ORF7b, ORF8, ORF9b, ORF14, Nucleocapsid (N), and ORF10.

SARS-CoV-2 is a positive-sense, single-stranded RNA (ssRNA), group IV virus. The genome was sequenced from the bronchoalveolar lavage fluid of a patient (Genbank: MN908947) and shared through the Global Initiative on Sharing All Influenza Data (GISAID) platform on 12th January 2020 (Wu et al., 2020). The ~30 k base pair genome is highly similar to the human SARS-CoV and bat CoV-SARS-like genomes with 14 open reading frames (ORFs) that encode structural, replication and non-structural accessory proteins, as depicted in Figure 1. Molecular modeling studies demonstrate that like SARS-CoV, SARS-CoV-2 is surrounded by a lipid bilayer membrane, containing structural membrane (M) and envelope (E) proteins that interact to form the viral envelope (Durrant et al., 2020). This layer also contains spike glycoproteins (S) that give the characteristic “corona” appearance of this family of viruses. The spike proteins bind specific host cell receptors to facilitate host cell attachment and entry (Graham and Baric, 2010). The nucleic acid-associated protein binds the RNA genome and forms the nucleocapsid (N). Other proteins include replication and non-structural accessory proteins that are listed in Table 1. Reports of different strains of SARS-CoV-2 suggest an early split from the SARS-CoV-2 lineage and/or the virus is mutating. Ongoing research provides insight into the unique and conserved features of the genome and proteome of SARS-CoV-2 to track mutations and generates evidence about the evolution of the virus (Phan, 2020; Wang et al., 2020). This is important as these changes may affect key structural and non-structural components of SARS-CoV-2 that can render some diagnostic tests ineffective or less sensitive and can also impact the selection of epitopes for the development of new tests.

**Table 1:** Proteins associated with the 14 ORFs of SARS-CoV.

**UniprotKB Entry**	**Protein**	**Gene**	**Function**
P0DTC1	Replicase polyprotein 1a (R1a)	ORF1a	Viral transcription/replication
P0DTD1	Replicase polyprotein 1ab (R1ab)	ORF1b	Viral transcription/replication, ribosomal frame shift
P0DTC2	Spike glycoprotein (S)	S	Attachment and host cell entry
P0DTC3	Protein 3a	ORF3a	Forms potassium-ion channel on the host cell membrane, and aids in virion assembly
P0DTC4	Envelope small membrane protein (E)	E	Virion assembly and morphogenesis
P0DTC5	Membrane protein (M)	M	Virion assembly and morphogenesis
P0DTC6	Non-structural protein 6	ORF6	Interferon antagonist
P0DTC7	Protein 7a (NS7A)	ORF7a	Activates the release of pro- inflammatory cytokines for viral pathogenesis
P0DTD8	Protein 7b (NS7B)	ORF7b	Structural and accessory protein
P0DTC8		ORF8 (different)	Unknown, but interacts with protein E
P0DTC9	Nucleoprotein (N)	N	Viral genome packaging, transcription, and virion assembly
P0DTD3	Uncharacterized protein 14	ORF9b	Unknown
P0DTD2	Protein 9b	ORF10	Unknown
A0A66DJA2	Hypothetical ORF10 protein	ORF14	Expression not known

The spread of SARS-CoV-2 is primarily by respiratory droplets that arise from individuals that harbor the virus. Symptomatic individuals with the disease are one source of virus, but a major public health concern is transmission by mildly ill or asymptomatic individuals during the incubation period. Rapid viral diagnostic testing for SARS-CoV-2 is critical to identify these individuals and facilitate the implementation of protective measures such as social distancing, quarantine and isolation that help to mitigate the spread of the virus in the community. The development of rapid and accurate tests that detect antibodies post-infection provide information about an individual's exposure to the virus and can be used to monitor the possibility of immunity, relapse or reinfection. This supports interventions to protect higher risk populations from developing more severe illness and can be used to investigate the efficacy of passive antibody therapies for COVID-19 infection. This review describes the available testing methods for SARS-CoV-2 and brings to light the importance of laboratory testing to control this disease and prepare for possible future disease threats.

## Detection of SARS-CoV-2 by Real Time Reverse-Transcriptase Polymerase Chain Reaction (RT-PCR)

RT-PCR detects the genetic material of SARS-CoV-2 to identify the virus and/or quantify viral load. Comparisons of the ssRNA genetic sequences of this virus have shown similarities to SARS-CoV and several bat coronaviruses (Lu et al., 2020). This detailed knowledge has allowed the rapid development of RT-PCR assays for SARS-CoV-2 using SARS-CoV and known CoVs as references.

### Sample Collection

Upper and lower respiratory samples are collected for detection of HCoV. Nasopharyngeal swabs are high priority specimens for SARS-CoV-2, and low priority specimens include oropharyngeal swabs, bronchoalveolar lavage, tracheal aspirates, and sputum (CDC, 2020a).

#### RNA Isolation

RNA is extracted from clinical specimens using approved viral isolation kits (Corman et al., 2020).

#### Real-Time RT-PCR

RNA is reverse transcribed to cDNA and subsequently amplified using a real-time quantitative PCR instrument. WHO announced various primer and probe sets for SARS-CoV-2 previously developed in China, Germany, Hong Kong, Japan, Thailand, and USA (World Health Organization, 2020b; Table 2). Primers targeting different sections of the virus genetic sequence including the envelope E gene, the RNA-dependent RNA polymerase (RdRp) gene, and the N gene (Chu et al., 2020; Corman et al., 2020; World Health Organization, 2020b). Targeting the E gene is reported for highest sensitivity, followed by the RdRp gene for confirmation (Corman et al., 2020). Some laboratories have multiplexed PCR tests consisting of multiple primer and probe sets located at different regions in the SARS-CoV-2 genome. These assays can be designed to contain primer sets targeting multiple genes simultaneously (RdRp/hel, S, N) (Chan et al., 2020b), or to detect different regions in a single target such as the N gene (U.F.A.D. Administration, 2020 Table 2). The use of multiplex assays is potentially beneficial as this can improve sensitivity in cases of loss or degradation of viral RNA during specimen collection and nucleic acid extraction, or in the event of mutation of the virus genome. These assays use *in vitro* synthesized RNA derived from transcripts (e.g., BetaCoV_Wuhan_WIV04_2019, GISAID Access number: EPI_ISL_402124) as positive controls and to generate standard curves. An internal control using RNAse P (RP) verifies the presence and quality of nucleic acid in samples and molecular grade nuclease-free water is used as a negative amplification control. A negative patient sample serves both as a negative extraction control to monitor cross contamination across samples and to validate test reagents.

**Table 2:** Table of primer and probe sequences for detecting SARS-CoV-2 genes.

**Country**	**Institute**	**Gene target**	**Sequence**	**References**
China	China CDC	ORF1ab	F: CCCTGTGGGTTTTACACTTAA	Ledsgaard et al., 2018
			R: ACGATTGTGCATCAGCTGA	
			P: 5′-FAM-CCGTCTGCGGTATGTGGAAAGGTTATGG-BHQ1-3′	
		N	F: GGGGAACTTCTCCTGCTAGAAT	
			R: CAGACATTTTGCTCTCAAGCTG	
			P: 5′-FAM-TTGCTGCTGCTTGACAGATT-TAMRA-3′	
Germany	Charité	RdRP	F2: 5′-GTGARATGGTCATGTGTGGCGG-3′	Chu et al., 2020
			R1: 5′-CARATGTTAAASACACTATTAGCATA-3′	
			P2: 5′-FAM-CAGGTGGAACCTCATCAGGAGATGC-BBQ-3′	
			P1: 5′-FAMCCAGGTGGWACRTCATCMGGTGATGC-BBQ-3′	
		E	F1: 5′-ACAGGTACGTTAATAGTTAATAGCGT-3′	
			R2: 5′-ATATTGCAGCAGTACGCACACA-3′	
			P1: 5′-FAM-ACACTAGCCATCCTTACTGCGCTTCG-BBQ-3′	
Hong Kong SAR	Hong Kong University	ORF1b	F: 5′-TGGGGYTTTACRGGTAACCT-3′	World Health Organization, 2020b
			R: 5′-AACRCGCTTAACAAAGCACTC-3′	
			P: 5′-FAM-TAGTTGTGATGCWATCATGACTAG-TAMRA-3′	
		N	F: 5′-TAATCAGACAAGGAACTGATTA-3′	
			R: 5′-CGAAGGTGTGACTTCCATG-3′	
			P: 5′-FAM-GCAAATTGTGCAATTTGCGG-TAMRA-3′	
Japan	National Institute of Infectious Diseases, Depart of Virology III	N	F: 5′-AAATTTTGGGGACCAGGAAC-3′	Nie et al., 2020
			R: 5′-TGGCAGCTGTGTAGGTCAAC-3′	
			P: 5′-FAM-ATGTCGCGCATTGGCATGGA-BHQ-3′	
Thailand	National Institute of Health	N	F: 5′-CGTTTGGTGGACCCTCAGAT-3′	Notomi et al., 2000
			R: 5′-CCCCACTGCGTTCTCCATT-3′	
			P: 5′-FAM-CAACTGGCAGTAACCABQH1-3′	
USA	US Center of Disease Control and Prevention	N	F: 5′-GAC CCC AAA ATC AGC GAA AT-3′	Li J. et al., 2018
			R: 5′-TCT GGT TAC TGC CAG TTG AAT CTG-3′	
			P: 5′-FAM-ACC CCG CAT TAC GTT TGG TGG ACC-BHQ1-3′	
			F: 5′-TTA CAA ACA TTG GCC GCA AA-3′	
			R: 5′-GCG CGA CAT TCC GAA GAA-3′	
			P: 5′-FAM-ACA ATT TGC CCC CAG CGC TTC AG-BHQ1-3′	
			F: 5′-GGG AGC CTT GAA TAC ACC AAA A-3′	
			R: 5′-TGT AGC ACG ATT GCA GCA TTG-3′	
			P: 5′-FAM-AYC ACA TTG GCA CCC GCA ATC CTG-BHQ1-3′	
			RP-F: 5′-AGA TTT GGA CCT GCG AGC G-3′	
			RP-R: 5′-GAG CGG CTG TCT CCA CAA GT-3′	
			RP-P: 5′-FAM – TTC TGA CCT GAA GGC TCT GCG CG – BHQ-1-3′	
France	Institut Pasteur	RdRP	F: 5′-ATGAGCTTAGTCCTGTTG-3′	Vincent et al., 2004
			R: 5′-CTCCCTTTGTTGTGTTGT-3′	
			P: 5′-AGATGTCTTGTGCTGCCGGTA [5′]HEX [3′]BHQ-1-3′	
			F: 5′-GGTAACTGGTATGATTTCG-3′	
			R: 5′-CTGGTCAAGGTTAATATAGG-3′	
			P: 5′-TCATACAAACCACGCCAGG [5′]FAM [3′]BHQ-1-3′	
		E	F: 5′-ACAGGTACGTTAATAGTTAATAGCGT-3′	
			R: 5′-ATATTGCAGCAGTACGCACACA-3′	
			P: 5′-ACACTAGCCATCCTTACTGCGCTTCG [5′]FAM [3′]BHQ-1-3′	

### Advantages

RT-PCR is the frontline diagnostic test for COVID-19 that is capable of analyzing thousands of specimens in a single day and shows a testing sensitivity of 95% (Corman et al., 2020). The anticipated limit of detection of the SARS-CoV-2 RT-PCR test is <10 copies/reaction (Chu et al., 2020) which allows early detection of low viral titers. Gene amplification indicates a positive result for the presence of SARS-CoV-2 RNA and should correlate with clinical observations, patient history, and epidemiological information.

### Disadvantages

False positive results could be generated by cross-reactivity of primers with nucleic acids arising from co-infection with other viruses or bacteria. In these cases, the agent detected may not be the definite cause of disease. Matching of the SARS-CoV-2 RT-PCR primers and probes using reliable libraries (e.g., BLAST) is necessary to ensure there is no homology with other CoVs like SARS-CoV from 2003 or other organisms such as *Staphylococcus aureus* and *Candida albicans*. False positives can also occur if reagents in a laboratory become contaminated, which is a major concern, particularly with the high volume of testing encountered during a pandemic. A negative patient sample is useful to identify this error in testing.

False-negative results could potentially arise from mutations occurring in the primer and probe target regions in the SARS-CoV-2 genome. Negative results do not preclude SARS-CoV-2 infection, and results should be validated with different primer sets against the same gene target and combined with patient history and other clinical data to accurately determine patient infection status.

### Key Logistics

Provisions for testing laboratories, the use of approved tests and validation of results with governing authorities to develop master protocols for use by multiple investigators must be in place to achieve rapid testing capacity. The output for number of tests per day and number of individuals tested per day relies on the laboratory capacity, trained staff, reagents, supplies and equipment. Large quantities of specific high-grade reagents are needed to perform tests and supplies can be quickly depleted in a pandemic. This impacts the turnaround time for RT-PCR diagnostic testing that ranges between 2 and 5 days. Strategies to rapidly scale up testing for novel HCoVs must be considered for future diagnostic testing.

## Detection of Antibodies Against SARS-CoV-2 Proteins by Enzyme Immunosorbent Assay (EIA)

EIA assays are diagnostic methods used to identify antibodies in patient blood sample or nasopharyngeal swabs. Enzyme-linked immunosorbent assays **(**ELISAs) for antibody detection against SARS-CoV-2 measure the host humoral response including IgM, IgG, and IgA to define previous exposure to the virus (Guo et al., 2020; Okba et al., 2020). IgM is the first immunoglobulin that is produced in response to an antigen and is primarily detected during the early onset of disease (3–7 days). IgG is the most abundant immunoglobulin that is produced in response to an antigen (7–25 days) and is maintained in the body after initial exposure and may have a protective role for acquired immunity. The IgA immunoglobulin plays a crucial role in the immune function of mucous membranes.

The SARS-CoV-2 S glycoprotein that mediates attachment and entry into cells is surface exposed and is a key target for the production of host neutralizing antibodies (Walls et al., 2016). This feature has made the S protein the focal target of antibody and vaccine development. The N protein in HCoVs functions as an antagonist of interferon (Kopecky-Bromberg et al., 2007; McBride et al., 2014) and viral encoded repressor (VSR) of RNA interference (RNAi) that facilitates viral replication, and is also a key target for antibody design (Leung et al., 2004). Recombinant antigens derived from the receptor binding domain of S protein (rS) as well as recombinant N protein (rN) are being developed as suitable diagnostic targets to detect IgM, IgG, and IgA antibodies. Dual detection of IgM/IgG and IgG/IgA immunoglobulins is under development for use in conjunction with nucleic acid detection for detecting active infection and to define previous exposure to SARS-CoV-2.

### Sample Collection

Systemic blood samples are collected from individuals for extraction of serum.

#### Enzyme-Linked Immunosorbent Assay (ELISA)

Purified rS or rN are immobilized to the surface of a multi-well-plate as capture antigens. Controls and inactivated SARS-CoV-2 serum samples are incubated with the antigen for SARS-CoV-2 antibody-antigen binding. A labeled secondary antibody-conjugate (e.g., horseradish peroxidase) is bound to the SARS-CoV antibodies for signal detection by substrate addition, and quantification.

### Advantages

Antibody tests provide the advantage of a simple method of detection of SARS-CoV-2 antibodies and are convenient to compare multiple samples from a single patient. Positive rates of detection for SARS-CoV-2 IgG in patients by ELISA measurements are 85.4% and 75.6–93.1% for IgM (Guo et al., 2020). Jin et al. (2020) reported sensitivities of serum IgM and IgG antibodies for detection were 48.1 and 88.9%, and specificities were 100 and 90.9% with the highest sensitivity for antibody tests recorded 2 weeks after first symptoms of disease. The lower IgM sensitivity may be because the IgM response occurs early then decreases and does not offer a strong detectable signal, while IgG signals may be more readily detected and present beyond 20 days. The incorporation of unique immunoglobulin labels may increase the sensitivity of rapid antibody tests for respiratory viruses (Li R. et al., 2018). Results from antibody testing could inform infection status and define previous exposure to SARS-CoV-2. Antibody detection is also used to identify recovered patients as human donors for the generation of convalescent patient serum or plasma as an investigational treatment for critically ill patients (Shen et al., 2020).

### Disadvantages

The results of SARS-CoV-2 antibody tests may vary by apparent disease periods by time after symptom onset as well as on the reliability of diagnostic assays. It is not yet known when IgM or IgG antibodies specific to the SARS-CoV-2 virus will become detectable during an infection, how long antibodies persist following infection and the extent of protection of neutralizing antibodies against subsequent infection with the virus.

The overall sensitivity and specificity indicate the possibility of false negatives and false positives in this testing method. Since the risk for recurrent infection with SARS-CoV-2 is not known for COVID-19, detection of one or two antibodies (IgM and/or IgG) does not necessarily guarantee immunity against reinfection. Negative results do not rule out SARS-CoV-2 infection, particularly in those who have been in contact with the virus and positive results may be due to past or present infection with SARS-CoV (Guo et al., 2020) and possibly non-SARS-CoV strains (Gaunt et al., 2010). It will be critical to conduct stringent evaluation of antibody diagnostic assays to determine the accuracy and reliability of results.

### Key Logistics

Recombinant systems are routinely used to express recombinant proteins to develop antibody assays. However, protein-expression systems can result in significant discrepancies between recombinant and native viral proteins. For example, the use of *E. coli* competent cells produces proteins that lack critical post-translational modifications in human cells (e.g., glycosylation) that can alter epitopes and protein conformation (Gupta and Shukla, 2018). Consequently, this can compromise sensitivity and specificity of antigens for diagnostic assays. The use of mammalian expression systems to express recombinant proteins will produce antigens with post-translation modifications that more closely resemble human native proteins (Bandaranayake and Almo, 2014) leading to higher sensitivity and specificity of assays.

Serological assays are currently under accelerated development for diagnosis of HCoV infections. Commercial reagents need to be validated by clinical trials using samples from patients with confirmed infections of SARS-CoV-2, and approved by the regulatory review process. Nonetheless, a rapid and sensitive platform for identification of antibody titers will also support screening to identify and minimize the risk of viral spread to others, as well as for epidemiological studies and vaccine evaluation studies. The US FDA allows the use of rapid antibody tests for SARS-CoV-2 under emergency use authorization (EUA). This expedites the assessment and optimization of these diagnostic tests, with the expectation that any test is sufficiently experimentally validated before it is made available to patients. If these tests do not provide accurate results, this can impair prevention efforts and delay appropriate treatment during the global pandemic response.

## Rapid Detection of SARS-CoV-2 by Lateral Flow Immunoassays (LFIA)

Several research laboratories have used the EIA platform to develop lateral flow immunoassays (LFIA) for the rapid qualitative detection of SARS-CoV. This is designed as a simple, portable diagnostic strip to measure either SARS-CoV-2 antibodies or antigens. As viral titers are often low in nasal swabs and serum or plasma, detection of antigens may be more challenging in comparison to detection of antibodies. Serological antigen assays can target S1 and S2 domains of the S protein that binds angiotensin-converting enzyme-2 (ACE-2), an integral transmembrane protein in the lung alveolar epithelium that serves as the initial attachment site for SARS-CoV-2, or N proteins.

### LFIA

The design of the lateral flow test is that of a strip/dipstick containing immobilized test reagents, enclosed in a cassette. Drops of a patient's blood are deposited on the strip which contains a coating of purified monoclonal antibody (mAb) or recombinant antigen that is localized at specific regions on a nitrocellulose membrane. The mAb targets a viral antigen; the recombinant antigen is recognized by antibodies that are present in infected patients. The strip also contains labeled detector antibodies that bind the same antigen. A positive antibody result indicates binding between the coating antigen and patient antibodies and binding by the detector antibody. This generates a colored signal. A positive antigen result indicates binding between the coating antibody and patient antigen.

#### Advantage

Two drops of blood are sufficient for detection of SARS-CoV-2 and antibodies by this method. This technique delivers results in ~15 min, and uses visual detection by the naked eye in comparison to RT-PCR (2–5 days). Detection of antibodies shows previous viral exposure while detection of antigens indicates active carriers of SARS-CoV-2 virus. The specificity and sensitivity of LIFAs are comparable for antibody and antigen assays.

#### Disadvantage

Tests to detect SARS-CoV-2 in patients by identifying viral antigens are more challenging to develop than tests to detect the neutralizing antibodies against SARS-CoV-2 (see below), as purified monoclonal antibodies must be generated against target antigens. Further, these assays need to be assessed and optimized using blood from infected patients.

#### Key Logistics

The rapid development of some antigens for assays are led by the use of “prototype” pathogens and *in silico* models of antibody–antigen interactions that are used to generate artificial antibody libraries (Shao et al., 2007). Antibody phage display technology can be applied to discover antibodies against antigens (Ledsgaard et al., 2018). These can be rapidly generated to produce prototypes of diagnostic tests for validation studies that expedite assessment and optimization, before the final commercial diagnostic kits are available. Integrating fast, portable tests with epidemiological surveillance will also provide quick and reliable information to public health authorities monitoring the spread of SARS-CoV-2.

## Serum Virus Neutralization Assay (SVN)

The SVN assay is a serological test that measures the ability of a patient's antibodies to neutralize infectivity of SARS-CoV-2 and attenuate infection. This assay is considered the most reliable for the assessment of protective antibody and can inform the use of convalescent plasma as a passive antibody therapy for COVID-19 infection particularly in severely ill patients. Although there is limited clinical data, early studies suggest that transfusion of convalescent plasma can suppress SARS-CoV-2 viral replication and protect an individual from infection (Guo et al., 2020; Shen et al., 2020). The SVN assay is not used for routine diagnosis but is frontline for this special indication.

### Sample Collection

Plasma is prepared from systemic blood samples collected from COVID-19 convalescent donors. Written informed consent is required from both the donor and recipient.

#### SVN

Several cell lines are suitable for SARS-CoV-2 transduction including Vero (monkey kidney cell line), Huh7 (human hepatoma cell line), 293T (human kidney cell line) (Nie et al., 2020). Serial dilutions of patient convalescent serum are added to known strains of virus (BetaCoV/Shenzhen/SZTH-003/2020 strain virus, GISAID access number: EPI_ISL_406594) (Shen et al., 2020). The mixture is inoculated into a susceptible cell monolayer and incubated for virus adsorption. The cytopathic effect can be measured by microscopic examination (Shen et al., 2020) after a 5-day incubation or fluorescence (Nie et al., 2020) or plaque formation, following 24 h of incubation. The neutralizing antibody titer is the highest dilution of serum that reduces activity of SARS-CoV-2.

### Advantages

The SVN assay is a highly robust and reproducible test that may be applied to detect SARS-CoV-2 neutralizing antibodies in convalescent plasma samples to identify the best candidates for treatment. Neutralizing activities along with viral load and antibody titers can be simultaneously monitored in paired plasma samples in patients receiving convalescence plasma, to establish algorithms for determining patient and donor factors that predict clinical efficacy.

### Disadvantages

The accessibility of the live SARS-CoV-2 strain is regulated, which limits the development of laboratory testing by SVN. While inexpensive, it is a manual assay and requires careful in-house standardization and quality control.

### Key Logistics

The preliminary case report of positive responses of 5 severely ill patients with COVID-19 who were treated in the Shenzhen Third People's Hospital, China, using plasma from recovered individuals was recently published (Shen et al., 2020). The convalescent plasma contained functional IgG and IgM anti–SARS-CoV-2 neutralizing antibodies that inhibited viral growth in cell cultures. Notably, the SNV assay reliably measured the increases in the patients' neutralizing antibody titers between 1 and 12 days after plasma transfusion. This study was not evaluated in a randomized clinical trial and there are limitations to the data interpretation. Nonetheless, these findings demonstrate the utility of the SVN assay for evaluating anti–SARS-CoV-2 neutralizing antibodies for future convalescent plasma assessment in more rigorous clinical investigations involving a larger cohort of patients with severe COVID-19 illness.

## Emerging Methods for Diagnosis of SARS-CoV-2

Methods for the rapid detection of nucleic acids are being used to develop applications in clinical diagnostics of SAR-CoV-2.

### Isothermal Nucleic Acid Amplification

This method amplifies DNA or RNA target sequence in a streamlined and exponential manner for detection, and in contrast to PCR, does not require thermal cycling. A wide variety of nucleic acid detection assays have been developed including loop-mediated isothermal amplification (LAMP), a single-tube technique for the amplification of DNA and reverse transcription-LAMP that combines reverse transcriptase and LAMP to detect RNA (RT-LAMP; Notomi et al., 2000), recombinase polymerase amplification (RPA; Li J. et al., 2018), helicase-dependent amplification (HDA; Vincent et al., 2004), strand displacement amplification (SDA; Walker et al., 1992), and nucleic acid sequence-based amplification (NASBA; Compton, 1991). These assays incorporate isothermal methods to enable primer binding followed by amplification using a polymerase with strand-displacement activity that separates the strand that is annealed to the target sequence for detection. Amplified gene products can be detected by photometry. Isothermal nucleic acid amplification is utilized in several commercial molecular diagnostic platforms and is considered the fastest available molecular laboratory and point-of-care test for the detection of novel SARS-CoV-2.

### RT-LAMP

The RT-LAMP method has been shown to effectively detect SARS-CoV-2 in clinical samples from individuals with COVID-19 (Yan et al., 2020). Multiple loop primers targeting the ORF1ab gene and the S gene were used for DNA strand displacement activity and target amplification that achieved detection of 20 copies/reaction and 200 copies/reaction, respectively. These results were comparable to RT-PCR amplification. The reported 100% sensitivity and 100% specificity and the mean time for detection was under 30 min, demonstrates this is a definitive testing method.

### RPA

This method detected total viral RNA derived from cell culture supernatant and 19 nasopharyngeal swab samples (8 positive and 11 negative) for SARS-CoV-2 (Behrmann et al., 2020). This approach integrates isothermal methods for reverse transcription followed by recombinase activity that mediates primer (targeting the N gene) binding to the homologous sequence in dsDNA. Subsequent amplification by polymerase mediated primer extension achieved 100% diagnostic sensitivity and specificity. This method offers potential advantages over RT-PCR for speed, scale and portability, allowing evidence-based clinical decisions to be made during a patient visit.

### CRISPR (Clustered Regularly Interspaced Short Palindromic Repeats)

The CRISPR assay functionality is being applied for detection of DNA or RNA using nucleic acid pre-amplification combined with CRISPR-Cas enzymology for specific recognition of sequences.

The CRISPR/Cas13a system is a recently discovered CRISPR-RNA (crRNA) guided detection method that is specific for RNA and is being applied for SARS-CoV-2 detection. A key feature of this approach is the Cas13a (formerly named C2c2) enzyme that recognizes and binds targeted RNAs in a sequence-specific manner followed by non-specific *trans*-endonuclease cleavage of non-targeted RNA (“collateral” cleavage) for signal amplification and nucleic acid detection. The Cas13a assay can be paired with target nucleic acid amplification for more sensitive results using an isothermal exponential amplification technique, most commonly RPA. This coupled technique is termed SHERLOCK (Specific High-Sensitivity Enzymatic Reporter unLOCKing) and allows fluorescence, colorimetric, lateral flow, and other readout approaches to enable the rapid detection of a variety of targets (Kellner et al., 2019).

### Cas13a Assay

Unlike *in vivo* CRISPR tools, the Cas13a protein must be recombinantly expressed and purified. The endonuclease activity of purified Cas13a uses crRNA targeting sequences in the S gene and ORF1ab in SARS-CoV-2 RNA. Target site-recognition activates *trans*-cleavage of reporter probes resulting in increases in fluorescence output signals and confirming the presence of viral RNA. Using synthetic SARS-CoV-2 the reported performance of this method for detection of target sequences is 20–200 aM (Kellner et al., 2019). The Cas13a/crRNA platform has been adapted for lateral-flow assays and could have wide applications as a SARS-CoV2 detector in both research and in the clinic. Assays can be designed as a paper dipstick test that delivers signals in 30–60 min using. This is a very promising technology and these positive advances in science offer immense hope for future disease control.

### Next Generation Sequencing

Next-generation sequencing (NGS) enables complete sequencing of the ~30,000 nucleotides of the SARS-CoV-2 genome. NGS provides a method for identification of SARS-CoV-2, for environmental monitoring and surveillance testing, while also providing insight into strain origin and viral evolution. Each sequence is deposited into the GISAID EpiCoVTM Database and to date, there are over 17,000 SARS-CoV-2 sequences from global NGS efforts.

#### Sample Preparation

RNA is extracted from clinical specimens, as for RT-PCR, and further purified to remove human cytoplasmic and ribosomal rRNA.

#### Library Preparation

RNA is fragmented followed by cDNA synthesis. Through the use of a set of highly specific, universal CoV primers, all genomic segments are amplified and the DNA amplicons are sequenced to deliver highly accurate SARS-CoV-2 typing in <24 h. Virus titer, efficiency of human rRNA depletion, and the number of reads per sample impact the number of virus-specific reads obtained and accurate coverage of the viral genome.

Collectively, global NGS data suggest that SARS-CoV-2 genome is relatively stable, although mutations are being identified in symptomatic individuals that are not present in the original strain in Wuhan, China. Two recent NGS studies report a large base pair deletion consisting of 81 nucleotides in SARS-CoV-2 ORF7a in a virus sample from a US patient (Holland et al., 2020), and point mutations that may suggest a more infectious strain of the virus than the original strain (Korber et al., 2020). The ORF7a gene encodes an accessory protein that is involved in viral infection and host cell death (Schaecher et al., 2007). These findings require investigation in other patient samples and to determine whether such mutations are selected in asymptomatic or symptomatic individuals. Although NGS is one of the most comprehensive approaches for identifying SARS-CoV-2, this method is relatively expensive, with multiple sample preparation steps and is not used for large-scale testing.

##### Biosafety

Regulating authorities provide interim guidance on the handling of specimens associated with SARS-CoV-2 (CDC, 2020b). Samples for testing can be performed in a BSL-2 laboratory with unidirectional airflow and BSL-3 precautions, and respiratory protection and a designated area for personal protective equipment changes are recommended. Isolation of SARS-CoV-2 in cell culture and initial characterization of viral agents recovered in cultures of patient samples should be conducted at Biosafety Level 3 (BSL-3), with regulatory approval and guidance.

## Conclusion

**Figure 2:**
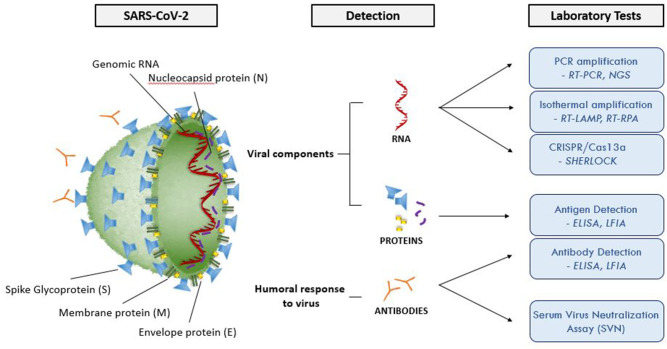
Molecular structure of SARS-CoV-2 and summary of the available laboratory tests and their target molecules. SARS-CoV-2 has a lipid bilayer membrane that contains Envelope (E) and Membrane (M) proteins that make up the envelope. Spike (S) glycoproteins project from the surface of the virion. Nucleocapsid protein (N) is composed of the protein that is associated with the viral genetic material. RT-PCR, reverse transcriptase polymerase chain reaction; EIA, enzyme immunoassay; LFIA, lateral flow immunoassay; SVNA, serum virus neutralization assay; INAA, isothermal nucleic acid amplification; CRISPR, clustered regularly interspaced short palindromic repeats; NGS, next generation sequencing; RT-LAMP, reverse transcriptase loop-mediated isothermal amplification; RPA, recombinase polymerase amplification.

Figure 2 and Table 3 summarize the main laboratory tests for detection of components of SARS-CoV-2 and the humoral response to the virus, and depict key features of these approaches. Given the public health emergency that the expanding COVID-19 outbreak presents, more widespread testing is needed to investigate the disease (e.g., prevalence in the population, severity in age groups), and to identify individuals who are infected but have few or no symptoms. Detailed epidemiological data sets will better establish the rates of severe infection and death among infected populations.

**Table 3:** Summary of main testing methods for COVID-19 highlighting the patient sample required for testing, material being tested, and key features.

**Method**	**Sample**	**Detected material**	**Key features**
RT-PCR	•Nasopharyngeal swab •Oropharyngeal swab •Bronchoalveolar lavage •Tracheal aspirates •Saliva	Viral RNA	•Duration: 2–5 days •Accuracy: High •Primary use: Gold standard diagnostic test •Cost: High (Reagents and Equipment) •Major limitations: Time and cross reactivity with other viruses (false positives)
EIA	•Blood •Nasopharyngeal swab	Antibodies/Antigens	•Duration: Hours •Accuracy: High •Primary use: Screening for exposure •Cost: High (Reagents and Equipment) •Major limitations: Cost, antigen detection is less accurate than RT-PCR
LFIA	Blood (finger stick) Saliva	Antibodies/Antigens	•Duration: Minutes •Accuracy: Lower than RT-PCR and EIA •Primary use: Rapid screening •Cost: Low •Major limitations: Lower accuracy particularly in antigen testing
SVN	Blood	Antibodies	•Duration: 5 days •Accuracy: High •Primary use: Detect neutralizing antibodies (convalescent plasma) •Cost: High •Major limitations: Duration
**Emerging Methods**			
Isothermalamplification •RT-LAMP •RT-RPA	Blood (finger stick)	Viral RNA	•Duration: Minutes (<30 min) •Accuracy: To be determined •Primary use: Rapid screening •Cost: Medium (Specific reagents) •Major limitations: Requires validation
CRISPR/Cas13a	Blood (finger stick)	Viral RNA	•Duration: Minutes •Accuracy: To be determined •Use: Rapid diagnosis •Cost: Low •Major limitations: Requires validation
NGS	Blood (finger stick)	Viral RNA	•Duration: Hours–days •Accuracy: High •Primary use: Genomic profiling of virus •Cost: High (Reagents and Equipment) •Major limitations: Cost, mainly used for genetic mapping rather than diagnostic

*RT-PCR, reverse transcriptase polymerase chain reaction; EIA, enzyme immunoassay; LFIA, lateral flow immunoassay; SVNA, serum virus neutralization assay; CRISPR, clustered regularly interspaced short palindromic repeats; NGS, next generation sequencing; RT-LAMP, reverse transcriptase loop-mediated isothermal amplification; RPA, recombinase polymerase amplification*.

Ongoing research is critical to optimize existing antibody tests to determine whether immunity prevents recurrent infection and to investigate the efficacy of passive antibody therapies for COVID-19 infection. The identification of novel disease biomarkers may be valuable for understanding what makes people susceptible to COVID-19 infection and in predicting the severity and progression of disease, Researchers could request approval to analyze stored samples of human blood or in animals that might be a natural reservoir of the virus. Specifically, guidance would be needed to direct blood and plasma collection centers to allow access of samples from COVID-19 patients.

The COVID-19 pandemic showcases how quickly information needs to be shared as responders address rapidly evolving situations. Establishing communication across laboratories worldwide helps to develop master protocols and establish reference panels for use by multiple investigators. This will aid in coordinating the collection and use of data, and regulatory infrastructure. Having a range of tests also puts less pressure on one manufacturer or supply chain, as different suppliers may use different materials. This could help alleviate difficult decisions to limit testing to the most vulnerable patients which can have great public health consequences.

## Author Contributions

RD'C and VS contributed to concept and writing of manuscript. AC contributed to writing, and prepared tables and figure for this manuscript.

## Conflict of Interest

The authors declare that the research was conducted in the absence of any commercial or financial relationships that could be construed as a potential conflict of interest.
